# Multidrug resistance, diagnostic challenges, and treatment gaps in *Pandoraea* infections: A review

**DOI:** 10.17305/bb.2025.13126

**Published:** 2025-10-23

**Authors:** Waiel S Halabi, Sulaiman Bani Abdel-Rahman, Hala Altarawneh, Rawan Altalhi, Loui A Ismaeel, Khulud A Alhazmi, Ohood S Alharbi, Malaz Gazzaz, Sarah Almuhayya, Turki M Alharthi, Bandar Hasan Saleh, Nabeel Hussain Alhussainy, Abdulaziz Alsaedi, Hatoon A Niyazi, Hanouf A Niyazi, Noha A Juma, Mona Abdulrahman, Karem Ibrahem

**Affiliations:** 1 Department of Optometry, Faculty of Applied Medical Sciences, University of Jeddah, Jeddah, Saudi Arabia; 2 Department of Microbiology and Pathology, Faculty of Medicine, Mutah University, Al-Karak, Jordan; 3 Department of Biological Sciences, College of Science, University of Jeddah, Jeddah, Saudi Arabia; 4 Department of Nursing, School of Applied Medical Sciences, University of Jeddah, Jeddah, Saudi Arabia; 5 Department of Microbiology and Parasitology, Faculty of Medicine, Umm Al-Qura University, Makkah, Saudi Arabia; 6 Pharmaceutical Practices Department, College of Pharmacy, Umm Al-Qura University, Makkah, Saudi Arabia; 7 Department of Clinical Laboratory Science, College of Applied Medical Sciences, King Saud University, Riyadh, Saudi Arabia; 8 Department of Clinical Laboratory Sciences, Faculty of Applied Medical Sciences, Umm Al-Qura University, Makkah, Saudi Arabia; 9 Department of Clinical Microbiology and Immunology, Faculty of Medicine, King Abdulaziz University, Jeddah, Saudi Arabia; 10 Department of Clinical Microbiology Laboratory, King Abdulaziz University Hospital, Jeddah, Saudi Arabia

**Keywords:** Pandoraea spp, multidrug-resistant pathogens, bacteriophages, antimicrobial peptides, combination antibiotic therapies

## Abstract

*Pandoraea* species are emerging multidrug-resistant (MDR) pathogens increasingly associated with respiratory tract infections, particularly in cystic fibrosis patients. Despite their growing clinical relevance, these bacteria are underrepresented in the scientific literature. This review aims to consolidate existing evidence regarding *Pandoraea* species as emerging MDR pathogens, with a focus on their taxonomy, diagnostic methodologies, antimicrobial resistance mechanisms, and treatment challenges. By identifying gaps in current therapeutic strategies and the limited clinical outcome data, this review underscores the necessity of advancing research into innovative interventions, such as bacteriophages, antimicrobial peptides (AMPs), and combination therapies, to enhance patient management and infection control. A comprehensive literature search was conducted using PubMed and Google Scholar, employing relevant keywords to identify case reports, clinical studies, and *in vitro* research related to *Pandoraea* infections, resistance mechanisms, and therapeutic strategies. Our findings reveal a significant lack of comprehensive data on therapeutic approaches, particularly concerning bacteriophages, AMPs, and combination antibiotic therapies. Furthermore, clinical data on treatment efficacy remain sparse, with the majority of evidence stemming from *in vitro* studies rather than real-world clinical settings. This review emphasizes the urgent need for further research to address these knowledge deficits and to develop effective therapeutic interventions against *Pandoraea* infections.

## Introduction

The *Pandoraea* genus consists of emerging Gram-negative, rod-shaped, obligately aerobic bacteria classified within the Burkholderiaceae family. First identified in 2000, this genus was established to reclassify certain species previously grouped under the Pseudomonas rRNA homology group II [1]. To date, 11 *Pandoraea* species have been identified and described, including *P. apista, P. pnomenusa, P. pulmonicola, P. sputorum, P. thiooxydans, P. norimbergensis, and P. oxalalivorans*, along with additional provisional species identified through molecular analyses [2, 3]. *Pandoraea* species are phylogenetically related to Ralstonia and Burkholderia spp., but are distinguished by 16S rRNA gene sequencing and multilocus sequence analysis [2]. These bacteria are ubiquitous in soil, water, and plant rhizospheres [4]; however, clinical isolates have increasingly been reported from respiratory secretions, blood cultures, and wound specimens [5]. The unique genomic features of *Pandoraea*, which include specific efflux pump families and β-lactamase genes, contribute to their environmental persistence and potential virulence [6].

Clinically, *Pandoraea* infections are predominantly associated with patients suffering from cystic fibrosis (CF), where they can colonize the respiratory tract and exacerbate chronic lung disease. Additionally, cases have been documented in immunocompromised and non-CF patients, including bloodstream infections, pneumonia, and bacteremia, underscoring their broader clinical significance. These bacteria are particularly concerning due to their distinctive multidrug-resistant (MDR) profiles, often exhibiting resistance to β-lactams, aminoglycosides, and carbapenems, along with variable susceptibility to agents such as imipenem, trimethoprim–sulfamethoxazole (TMS), and tetracyclines [5].

Despite their increasing clinical relevance, knowledge about *Pandoraea* remains limited, primarily derived from isolated case reports or small series. Investigating these pathogens is essential for enhancing clinical recognition, guiding infection control measures, and identifying novel therapeutic options. This review aims to provide a comprehensive overview of *Pandoraea* infections, focusing on their taxonomy, diagnostic challenges, antimicrobial resistance (AMR) patterns, and current therapeutic strategies. In doing so, it seeks to illuminate existing knowledge gaps, particularly the scarcity of clinical data on treatment efficacy, and to emphasize the urgent need for research into novel strategies such as bacteriophages, antimicrobial peptides (AMPs), and combination antibiotic therapies to improve the management of these emerging MDR pathogens.

## Methods

A structured literature search was conducted to identify publications regarding *Pandoraea* species. The search was performed in PubMed and Google Scholar using keywords including “*Pandoraea*,” “virulence,” “pathogenesis,” “epidemiology,” “antimicrobial resistance,” “treatment,” “phage therapy,” “antimicrobial peptides,” “combination therapy,” and “novel therapy.” No restrictions were applied regarding publication date or study type, given the limited literature available on *Pandoraea* infections. Inclusion criteria encompassed case reports, clinical studies, and *in vitro* research addressing taxonomy, identification, resistance mechanisms, and therapeutic strategies. Studies lacking primary data or relevance to human infections were excluded. Articles were screened by title and abstract, followed by a full-text review to extract key findings, with quality assessed based on study type and methodological rigor. This approach ensured a comprehensive synthesis of the existing evidence on *Pandoraea* infections.

## Epidemiology

Given the limited number of reported *Pandoraea* species infections, accurately defining their epidemiological profile remains challenging [7]. The majority of cases involved male patients, with a mean age of 42.08 years. Notably, most reported cases originated from European countries, while only 27.58% were documented in Asia and the Americas, and just 10.34% in Oceania. The observable predominance in Europe may reflect more effective surveillance systems or a higher incidence of CF within the population [8]. Conversely, the low incidence in Asia (specifically in China and India) and the absence of reports from Africa suggest that *Pandoraea* infections may not be strongly correlated with socioeconomic or environmental factors [5, 9]. Nonetheless, the overall limited data and high potential for misdiagnosis impede the ability to draw definitive epidemiological conclusions about the global distribution and burden of *Pandoraea* infections. The primary risk factor is CF, where *Pandoraea* colonization occurs in up to 5% of patients, often co-colonizing with Pseudomonas aeruginosa and Candida spp. [5]. Additional at-risk populations include individuals with chronic pulmonary conditions, hematologic malignancies, organ transplant recipients, and patients with implanted medical devices [5]. Outbreaks associated with contaminated hospital water sources and bronchoscopes have been documented, highlighting the potential for nosocomial transmission [5].

An outbreak of *Pandoraea pulmonicola* was reported in a CF center, affecting 6 out of 243 patients. Identification techniques included ARDRA, MALDI-TOF MS, and 16S rDNA sequencing, with PFGE validating clonal dissemination. The bacteria, presumably transmitted through droplets due to deficiencies in infection control, exhibited resistance to multiple antibiotics while remaining sensitive to TMS. All patients were co-colonized with *Pseudomonas aeruginosa*, and *P. pulmonicola* colonization became chronic. Three patients died, emphasizing the organism’s transmissibility and the necessity for stringent infection control measures [10].

In another case, a 46-year-old female without CF, suffering from injuries and burns, developed sepsis caused by *P. sputorum*, confirmed through 16S rRNA PCR and MALDI-TOF MS. The isolate displayed resistance to meropenem but was susceptible to imipenem and other specific antibiotics. Following targeted therapy and supportive care, the patient recovered. This case highlights the emerging virulence and resistance profile of *Pandoraea* species, with imipenem as a potential early therapeutic option [11]. This report is significant for clinicians managing rare MDR Gram-negative infections, particularly in trauma and non-CF patients. However, it underscores that *Pandoraea sputorum* infections are exceptionally uncommon in non-CF individuals. Additionally, the lack of comparisons with other reported cases or control groups limits the ability to ascertain whether the clinical presentation and treatment response observed here are unique or representative. Finally, the focus is restricted to short-term outcomes—patient discharge after two months—without long-term follow-up data to evaluate recurrence or delayed complications [11].

Despite the presence of *Pandoraea* species, an epidemic involving 24 non-CF patients occurred in two German hospitals between July 2019 and December 2021. Most patients were critically ill and had undergone surgery or received prior antibiotics. Genomic analysis identified a clonal strain, *Pandoraea* commovens LB-19-202-79, resistant to many antibiotics but susceptible to ampicillin/sulbactam, imipenem, and TMS. This outbreak underscores the potential for *Pandoraea* to spread in non-CF settings, emphasizing the need for increased clinical and microbiological awareness [12].

Although *Pandoraea* infections are infrequent, certain factors appear to elevate the risk. Individuals with CF are predominantly affected, as the bacteria are commonly found in their lungs. However, infections have also been observed in non-CF individuals, especially among those who are critically ill or hospitalized for extended periods [13]. Patients in the ICU, those on ventilators, or individuals who have recently undergone surgery or suffered major trauma—such as burns—are particularly vulnerable [1, 5]. The use of broad-spectrum antibiotics may exacerbate the situation by depleting normal flora, allowing *Pandoraea* to proliferate. In several instances, infections have been linked to hospital outbreaks, likely due to lapses in infection control measures [14]. Individuals with significant underlying health issues, particularly those with chronic pulmonary disorders or who have received lung transplants, may be at increased risk for *Pandoraea* infections.

A 30-year-old man developed sepsis due to *Pandoraea pnomenusa* following a lung transplant and ultimately succumbed, highlighting the considerable risk this pathogen poses to immunocompromised individuals. *P. pnomenusa*, a rare MDR Gram-negative bacterium, is typically resistant to most β-lactams and aminoglycosides, with imipenem often remaining effective; however, resistance patterns vary and necessitate *in vitro* testing. Treatment is particularly challenging due to its resistance profile and the absence of standardized guidelines, with empirical therapy often involving carbapenems (especially imipenem), cephalosporins, and TMS [15]. Outcomes are frequently poor, with high mortality rates observed in cases of sepsis and multi-organ failure following transplantation. However, the available data are limited to case reports and small series, making generalization difficult, and misidentification is common without advanced diagnostic tools. This case underscores the urgent need for rapid and accurate identification of *Pandoraea* species and tailored therapeutic strategies for transplant and other immunocompromised patients [15].

## Pathogenesis

**Figure 1.**
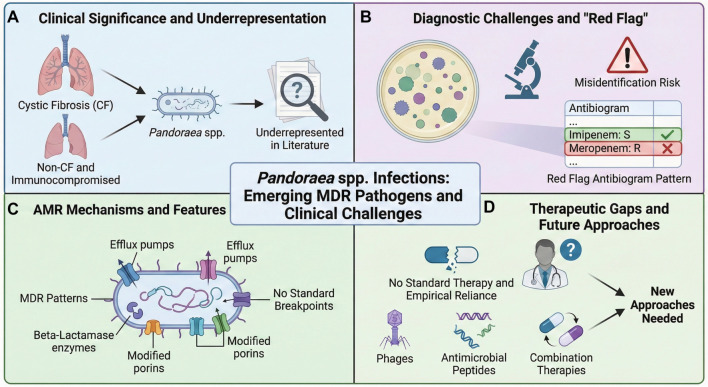
**Graphical overview of challenges associated with emerging multi-drug resistant (MDR) *Pandoraea spp.* infections.** This summary highlights key aspects of the pathogen's clinical and microbiological profile. (A) Clinical Significance and Underrepresentation: Identifies primary at-risk populations, specifically cystic fibrosis (CF) patients and non-CF immunocompromised individuals, noting that these pathogens remain under-researched in current literature. (B) Diagnostic Challenges and “Red Flag“: Illustrates laboratory difficulties, including the high risk of species misidentification. It emphasizes a characteristic “red flag” antibiogram pattern---susceptibility (S) to imipenem concurrent with resistance (R) to meropenem---that aids in identification. (C) AMR Mechanisms and Features: Depicts key intrinsic and acquired resistance mechanisms (efflux pumps, beta-lactamases, modified porins) contributing to MDR patterns, alongside the challenge posed by the absence of standardized antimicrobial susceptibility testing breakpoints. (D) Therapeutic Gaps and Future Approaches: Highlights the lack of established treatment regimens leading to reliance on empirical therapy, and outlines urgent needs for novel therapeutic strategies such as bacteriophages, antimicrobial peptides, and combination therapies.

The pathogenesis of *Pandoraea* infections is not yet fully understood, but it has been suggested that these bacteria act as opportunistic pathogens [5, 10], primarily affecting individuals who are already vulnerable, such as those with CF, weakened immune systems, or serious illnesses requiring ICU care [12, 13, 16] (Figure 1). They can be challenging to identify correctly, often being mistaken for other bacteria like *Burkholderia* or *Ralstonia*, which can delay diagnosis and treatment [17]. *Pandoraea* are known for their ability to form biofilms, particularly on medical devices such as catheters and ventilators, which facilitates their evasion of the immune system and resistance to antibiotics [8, 18]. The genome of *Pandoraea* sp. XY-2 contains genes likely involved in producing exopolysaccharides, including proteins such as PelF and PelG, which aid in the formation of protective biofilm layers. Additionally, genes like CdgC and LeuO are believed to assist in biofilm construction—slimy structures that enable survival in harsh conditions and resistance to antibiotics [18]. Furthermore, they often exhibit resistance to multiple antibiotics, including meropenem, complicating treatment [19]. In severe cases, particularly among immunocompromised patients, the bacteria can enter the bloodstream and trigger a robust inflammatory response, which may lead to sepsis or organ failure [10, 11, 20]. Overall, these infections primarily occur in individuals with compromised health, and their ability to resist treatment and evade detection poses significant challenges in hospital settings [21].

*Pandoraea* infections are generally acquired in hospital environments, particularly among critically ill patients or those undergoing intensive treatment [5, 12]. They commonly affect individuals who have recently undergone surgery, are on ventilators, or have medical devices such as catheters or central lines, which can serve as entry points for the bacteria [5, 22]. Infections may disseminate among patients if infection control protocols, including rigorous hand hygiene and equipment sterilization, are not strictly followed [5, 23]. In some cases, patients may initially become colonized with *Pandoraea*, particularly in the lungs, later developing an infection if their immune system is compromised or if they are treated with broad-spectrum antibiotics [5, 16, 22].

A 44-year-old male patient with a history of multiple trauma underwent evacuation of traumatic intracranial hematomas followed by decompressive craniectomy. During his hospital stay, *Pandoraea apista* was isolated from sputum samples and identified via MALDI-TOF MS. Antimicrobial susceptibility testing revealed a MDR pattern, with resistance to meropenem and variable susceptibility to other agents. The patient was treated with meropenem and vancomycin; however, despite therapy, his clinical status deteriorated, leading to his death. This case illustrates the challenges of managing severe infections caused by *Pandoraea apista* in critically ill patients, particularly given its unpredictable and resistant susceptibility profile [23]. This study is limited by its focus on a single patient, restricting the ability to generalize findings or draw firm conclusions about *Pandoraea apista* infections in non-CF patients. The pathogenic role and clinical significance of *Pandoraea* species in non-CF contexts remain unclear, as acknowledged by the authors. Furthermore, the lack of comparative analysis with other reported cases or organisms, combined with insufficient long-term follow-up, diminishes the report’s value in understanding patient outcomes and guiding management strategies. Although the study emphasizes the highly variable and MDR susceptibility profiles of *Pandoraea*, it does not provide systematic data on treatment effectiveness [23].

Lower respiratory tract involvement is predominant in respiratory infections, particularly among CF patients, manifesting as increased cough, sputum production, and deterioration in lung function [24]. Radiographically, *Pandoraea* infections may mimic Pseudomonas pneumonia, presenting with infiltrates and bronchiectasis [25, 26]. Acute exacerbations often necessitate hospitalization and intravenous antibiotics [27]. Bacteraemia and sepsis from *Pandoraea* bloodstream infections, though rare, have been documented in patients with central venous catheters, malignancies, or post-surgical infections [11]. Clinical features include fever, hypotension, and elevated inflammatory markers [28]. Other manifestations, including infective endocarditis, osteomyelitis, urinary tract infections, and wound infections, have been reported, albeit in limited numbers [5]. These infections often involve implanted devices or surgical wounds and require combined medical and surgical management [29]. Additionally, *Pandoraea* expresses lipopolysaccharides (LPS), which activate host immune responses. This response largely depends on the specific structure of the lipid A component of LPS. A recent analysis of a chronic strain of *P. pulmonicola* (RL 8228), known for its high virulence, revealed a smooth-type LPS with hypoacylated lipid A variants. These include rare features such as 2-hydroxylation of acyl chains and additional glucosamine substitutions on phosphate groups, providing new insights into the inflammation-inducing properties of this pathogen [30].

## AMR

Currently, there are no standardized antibiotic susceptibility breakpoints defined for *Pandoraea* species (Figure 1). These bacteria are generally classified as MDR, often exhibiting significant resistance to β-lactams and aminoglycosides. Recognizing their unique resistance profiles is critical for timely and effective infection control measures [1, 5]. *Pandoraea sputorum*, frequently isolated from CF patients, was first identified in Japan from an elderly male. Whole-genome sequencing of strain THI4931 revealed resistance to multiple antibiotics, including β-lactams, aminoglycosides, colistin, and polymyxin B, while remaining susceptible to aztreonam, imipenem, and minocycline. The genome harbored *AmpC* and *OXA-62* β-lactamase genes, underscoring the potential risk posed by *P. sputorum* in vulnerable hospitalized patients [31].

A separate study reported the complete sequences of eight *Pandoraea* plasmids for the first time, revealing unique features absent from public databases. Some plasmids lacked common replication and segregation genes, suggesting reliance on host interactions for maintenance. Notably, several plasmids carried toxin-antitoxin systems and conjugation genes, which may facilitate persistence and dissemination. These plasmids also contained virulence and antibiotic resistance genes, emphasizing the potential of *Pandoraea* spp. as emerging opportunistic pathogens [32]. A case was documented involving a patient with no history of CF who developed *P. sputorum* sepsis following multiple traumatic injuries and burns from a brick kiln collapse. The strain exhibited resistance to numerous antibiotics, including meropenem, quinolones, and aminoglycosides, but remained sensitive to imipenem, tetracyclines, and ampicillin/sulbactam. This resistance may be linked to the bacteria’s ability to form biofilms and produce carbapenemases, highlighting the importance of early identification and targeted therapy, as *P. sputorum* can pose a significant threat even in patients without underlying conditions [11].

Although the study predominantly focused on *Pseudomonas aeruginosa*, similar resistance mechanisms may be present in *Pandoraea* species (Figure 1). These mechanisms include the production of extended-spectrum beta-lactamases, enzymes that modify aminoglycosides, mutations in DNA gyrase and topoisomerase, loss of the OprD2 porin protein, and overactive efflux pumps. Collectively, these factors likely contribute to the high levels of multidrug resistance observed in *Pandoraea* [33]. Notably, *Pandoraea* species have been found to produce novel siderophores—pandorabactin A and B—through a conserved NRPS gene cluster (pan). These molecules, identified through genome mining and metabolite analysis, exhibit iron-chelating properties. Functional assays demonstrated that pandorabactins deplete iron and exhibit antibacterial activity against CF-associated lung pathogens, such as *Pseudomonas*, *Mycobacterium*, and *Stenotrophomonas*. Metagenomic analysis further associates the pan gene’s presence with microbial patterns in CF lung samples, illuminating *Pandoraea’s* virulence mechanisms and its role in shaping lung microbiota through iron competition [34] (Table 1).

**Table 1:** Key resistance features of *Pandoraea* spp.

**Aspect**	**Key findings**	**References**
Breakpoints	No defined standards; MDR common	[1, 5]
Resistance	Resistant to β-lactams, aminoglycosides, colistin; susceptible to imipenem, TMS, minocycline, aztreonam	[11, 31]
Resistance genes	*ampC*, *OXA-62*; plasmid-borne genes	[31, 32]
Plasmids	Unique, with toxin–antitoxin and conjugation genes	[32]
Mechanisms	Biofilm, carbapenemases, efflux, porin loss	[11, 33]
Metabolites	Pandorabactin siderophores (iron competition)	[34]

Abbreviations: MDR: Multidrug-resistant; TMS: Trimethoprim–sulfamethoxazole; β-lactams: Beta-lactam antibiotics; *ampC*: AmpC β-lactamase gene; OXA-62: Oxacillinase-62.

## Microbiology and identification

The accurate identification of *Pandoraea* species presents a significant challenge in clinical microbiology, primarily due to the diagnostic limitations of many standard hospital laboratories. Numerous clinical settings continue to rely on traditional biochemical techniques or commercial identification technologies, which may lack the necessary sensitivity or specificity to accurately distinguish rare or emerging infections. Advanced molecular techniques, such as 16S rRNA gene sequencing, whole-genome sequencing, or matrix-assisted laser desorption/ionization time-of-flight mass spectrometry (MALDI-TOF MS), have proven essential for the accurate classification of non-fermentative Gram-negative bacilli like *Pandoraea* [35]. However, these tools are often unavailable in resource-limited environments due to their costs, the need for technical expertise, and infrastructure demands.

Compounding these challenges, many commercial identification databases used in automated systems either exclude *Pandoraea* species altogether or contain incomplete reference profiles, leading to misidentification or ambiguous results [35]. This underscores the critical need for broader implementation of molecular diagnostics and ongoing updates of microbial databases to enhance the detection and characterization of rare pathogens like *Pandoraea* in clinical settings [35]. Advances in genetic techniques have significantly increased the identification of previously unrecognized microorganisms, thereby enhancing diagnostic accuracy, particularly for infections caused by organisms often overlooked by conventional microbiological methods [5, 36]. Conventional systems frequently misidentify *Pandoraea* as *Burkholderia* or *Ralstonia* [15]. Accurate identification of microorganisms typically relies on advanced microbiological methods, with 16S rRNA gene sequencing being one of the most reliable tools available [37].

*Pandoraea* spp. can be cultivated on conventional bacteriological media, such as tryptic soy agar, where they typically form cream-colored, circular, convex colonies with smooth, entire margins, approximately 1–2 mm in diameter [38].

A 9-year-old boy with CF, chronically colonized by *Pseudomonas aeruginosa* and *Staphylococcus aureus*, was diagnosed with a *Pandoraea sputorum* infection, which was initially misidentified but later confirmed using 16S rRNA sequencing and MALDI-TOF MS. The isolate exhibited a MDR profile, showing susceptibility only to imipenem and TMS. The patient was treated with both agents; however, *P. sputorum* was not eradicated, and chronic colonization persisted, contributing to progressive lung function decline. Despite this, his clinical condition improved following an adjustment in therapy to a combination of imipenem, amikacin, and colistin [37]. This report, the first documented case in Argentina, underscores the diagnostic challenges posed by *Pandoraea* species, the limited treatment options available, and the difficulty in attributing outcomes due to co-infection with other pathogens. While the single-case nature restricts generalizability, the case highlights the importance of accurate identification and the urgent need for further studies to clarify the pathogenic role and management strategies for *P. sputorum* in CF patients [37] (Table 2).

**Table 2:** Summary of reported clinical cases of *Pandoraea* infections, including treatment approaches, outcomes, and key clinical points

**Case**	**Treatment**	**Outcome**	**Key point**	**Ref.**
46F, non-CF, burns (*P. sputorum*)	Targeted therapy + supportive care	Recovered, discharged after 2 months	Rare non-CF case; imipenem effective	[11]
30M, lung transplant (*P. pnomenusa*)	Empirical carbapenems, cephalosporins, TMS	Died (sepsis, multi-organ failure)	High mortality in transplant pts; rapid ID crucial	[15]
44M, trauma (*P. apista*)	Meropenem + vancomycin	Died (clinical deterioration)	Poor prognosis; MDR, variable susceptibility	[23]
9M, CF (*P. sputorum*)	Imipenem + TMS (later with amikacin, colistin)	Colonization persisted; clinical improvement	Diagnostic challenge; first Argentinian CF case	[37]

These reports highlight the emerging clinical significance of multidrug-resistant *Pandoraea* species, their variable susceptibility profiles, and challenges in management. Abbreviations: CF: Cystic fibrosis; F: Female; M: Male; MDR: Multidrug-resistant; TMS: Trimethoprim–sulfamethoxazole; pts: Patients; ID: Identification.

Timely identification of *Pandoraea* infections necessitates heightened clinical awareness and microbiological vigilance, particularly due to the organism’s rarity and diagnostic challenges [15]. A key feature that facilitates its detection is its unique AMR profile, notably concerning carbapenems. *Pandoraea* species typically exhibit a distinctive resistance pattern characterized by susceptibility to imipenem while demonstrating resistance to meropenem—traits not commonly observed in other non-fermenting Gram-negative bacilli. This atypical carbapenem susceptibility, combined with broader multidrug resistance, serves as a critical diagnostic clue and highlights the need for accurate antimicrobial susceptibility testing to inform appropriate therapy [15, 39].

Precise identification of *Pandoraea* species often requires advanced microbiological techniques, particularly given the limitations of conventional diagnostic tools. Among these, matrix-assisted laser desorption/ionization time-of-flight mass spectrometry (MALDI-TOF MS) and 16S rRNA gene sequencing are the most reliable. In this review, MALDI-TOF emerged as the most frequently employed technique, closely followed by 16S rRNA sequencing [37]. In numerous cases, both methods were utilized in tandem to confirm diagnoses. For example, a study by Martina et al. demonstrated the effectiveness of combining 16S rRNA sequencing with mass spectrometry to accurately identify *P. sputorum*, a pathogen that had been misdiagnosed using standard laboratory methods [37, 40]. This underscores the importance of integrating multiple advanced technologies for the accurate detection of rare or atypical pathogens. Despite the efficacy of advanced molecular tools, their high cost and limited availability in many clinical laboratories necessitate reliance on distinctive biochemical and cultural characteristics to identify *Pandoraea* species. Supporting this approach, Jorgensen et al. employed pulsed-field gel electrophoresis (PFGE) for an epidemiological investigation of *P. apista* isolates, illustrating the value of conventional techniques in the absence of high-end molecular diagnostics [37, 40].

To assess the differentiation among *Pandoraea* species, restriction fragment length polymorphism (RFLP) analysis and direct sequencing of the gyrB gene were conducted on 67 isolates. Distinct RFLP patterns specific to each species were observed following the digestion of PCR-amplified *gyrB* gene with MspI, and these groupings were validated through sequencing of selected representative strains. The findings underscore the effectiveness of *gyrB*-based RFLP and sequencing as reliable tools for species-level identification of *Pandoraea* [41]. Additionally, the study suggests that improved primer sets could enhance the amplification and application of the *gyrB* gene in broader β-Proteobacteria taxonomy. Importantly, the classification of *Pandoraea* genomospecies 2 may require reevaluation based on genetic data [42]. Recently, a study reported the isolation of *Ralstonia* and *Pandoraea* species from respiratory cultures of CF patients [41]. Utilizing ribosomal DNA restriction analysis (ARDRA) for species differentiation within *Ralstonia* and *Pandoraea*, researchers compared restriction profiles of reference strains using six enzymes previously validated for *Burkholderia* identification. ARDRA successfully differentiated all tested *Ralstonia* species and *Pandoraea norimbergensis*, but was less effective at distinguishing *P. pnomenusa*, *P. sputorum*, *P. pulmonicola*, and *P. apista* at the species level [41]. Early diagnosis is crucial to prevent delays in initiating suitable therapy, which can significantly affect patient outcomes.

## Treatment

Currently, there are no established treatment protocols for *Pandoraea* infections, and therapy is often initiated empirically while awaiting susceptibility test results. The lack of explicit guidelines is likely due to the limited availability of public data globally. Treatment outcomes are variable, with higher mortality rates observed, often dependent on factors such as the infection site and the patient’s overall health status [5]. The management of *Pandoraea* infections remains largely unclear, contributing to ongoing diagnostic and therapeutic challenges. Infections caused by *Pandoraea* in non-CF patients may be underreported or misidentified, emphasizing the need for improved detection and a deeper understanding of these emerging pathogens [22]. Furthermore, the treatment of infections caused by *Pandoraea* species is particularly challenging due to their resistance to multiple antibiotics [22]. Carbapenems, especially imipenem, appear to be a viable treatment option for severe *Pandoraea* infections, as the majority of isolates show resistance to this antibiotic. Imipenem may provide a more effective therapeutic alternative compared to other antimicrobials, particularly in the absence of established treatment guidelines. TMS has also been commonly used in managing *Pandoraea* infections, with aminoglycosides and quinolones considered in certain cases [5]. However, due to the variable and often MDR nature of *Pandoraea* species, antibiotic selection should be guided by *in vitro* susceptibility testing to ensure effective and targeted therapy [5].

The antibiotic susceptibility profile of *Pandoraea* species isolated from blood and wound secretion samples in our case highlights a pattern of multidrug resistance. Across all samples, the isolates demonstrated resistance to a broad range of β-lactam antibiotics, including piperacillin, piperacillin/tazobactam, cefazolin, cefuroxime (both sodium and axetil forms), cefotetan, ceftazidime, cefepime, and aztreonam. Resistance was also noted against aminoglycosides (amikacin, gentamicin, and tobramycin) and fluoroquinolones (ciprofloxacin and levofloxacin), with only intermediate susceptibility to ceftriaxone and levofloxacin in two samples [11]. Notably, the isolates were uniformly susceptible to TMS and imipenem, while exhibiting resistance to meropenem, consistent with the known unique resistance profile of *Pandoraea* species (Figure 1). Additionally, ampicillin/sulbactam and minocycline demonstrated favorable activity, indicating their potential as alternative treatment options. This susceptibility pattern emphasizes the importance of accurate and timely antimicrobial susceptibility testing to inform effective therapy for *Pandoraea* infections [11].

A study demonstrated the *in vitro* efficacy of various β-lactam–β-lactamase inhibitor combinations, including ceftazidime-avibactam, ceftolozane-tazobactam, meropenem-vaborbactam, and piperacillin-tazobactam, as well as 11 additional antibiotics against 420 clinical isolates of *Burkholderia*, *Achromobacter*, Stenotrophomonas, and *Pandoraea* species [43]. Eighty-nine percent of these isolates were recovered from respiratory samples of individuals with CF. Among the newer combination therapies, meropenem-vaborbactam exhibited the strongest activity, particularly against *Burkholderia* and *Achromobacter* strains, including those with MDR and extensively drug-resistant (XDR) profiles [43]. However, none of the newer β-lactam–β-lactamase inhibitor combinations demonstrated improved efficacy over older agents when tested against *Stenotrophomonas maltophilia* and *Pandoraea* species, highlighting the persistent challenge of treating infections caused by these organisms [43].

There is a notable deficiency in detailed clinical data regarding the effectiveness of antimicrobial agents against *Pandoraea* infections. While some *in vitro* studies suggest potential activity, real-world evidence from clinical cases remains limited. Consequently, making confident therapeutic recommendations for *Pandoraea* infections based solely on existing data is challenging. There is an urgent need for targeted research, including clinical trials and case studies, to better understand the therapeutic possibilities and limitations of these antibiotics in treating *Pandoraea* species [5, 11, 22, 42] (Table 3).

**Table 3:** Summary of treatment and antimicrobial susceptibility in *Pandoraea* infections

**Aspect**	**Key findings**	**References**
Treatment guidelines	No standard therapy; empirical use common; outcomes vary	[5, 22]
Main effective agents	Imipenem, trimethoprim/sulfamethoxazole (TMS) most reliable	[5, 11, 22]
Alternative agents	Ampicillin/sulbactam, minocycline sometimes active	[11]
Ineffective agents	Resistance to most β-lactams, aminoglycosides, fluoroquinolones, meropenem	[11]
Challenges	MDR patterns, frequent misidentification, lack of clinical data	[5, 11, 22, 42]

Abbreviations: TMS: Trimethoprim/sulfamethoxazole; MDR: Multidrug-resistant.

## Discussion

### Future approaches

This study highlights a critical gap in the scientific literature concerning effective strategies to combat *Pandoraea* infections. Currently, none of the available reviews have addressed the isolation, characterization, or therapeutic application of bacteriophages specific to *Pandoraea* spp., representing a promising yet unexplored area. Additionally, there is a significant lack of studies evaluating the efficacy of AMPs or combination antibiotic therapies against this emerging pathogen. This limited evidence base underscores the urgent need for targeted research to develop innovative and effective treatment strategies.

Particularly, phage therapy should be investigated not only for its therapeutic potential but also for its role in mitigating biofilm-associated resistance, a hallmark of *Pandoraea* spp. AMPs also merit further investigation due to their unique mechanisms for disrupting bacterial membranes and their lower likelihood of inducing resistance compared to conventional drugs [44, 45]. Exploring rationally designed combination therapies could provide dual benefits—enhancing antimicrobial efficacy while simultaneously limiting resistance development. Furthermore, integrating genomic and transcriptomic studies could help identify potential molecular targets and resistance mechanisms, thereby guiding the design of novel interventions. Establishing international collaborative networks and multicenter studies will be essential for generating robust clinical evidence and translating these experimental strategies into viable therapeutic options [44, 45].

Bacteriophages and their derivatives, such as endolysins, play a crucial role in combating biofilms by degrading the extracellular matrix and directly killing embedded bacteria. These phages produce enzymes like depolymerases and endolysins that break down biofilm polysaccharides and peptidoglycan, enhancing biofilm penetration and bacterial eradication [44, 45]. Moreover, phage-antibiotic combinations and phage cocktails have demonstrated superior efficacy, as they not only synergistically disrupt biofilms but also help prevent or delay the development of resistance [44, 45]. For instance, phage vB_C4, which targets *Aeromonas veronii*, has shown remarkable effectiveness when combined with antibiotics, leading to significant removal of mature biofilms while simultaneously reducing the emergence of phage-resistant bacterial populations [44]. These studies underscore the potential of bacteriophage therapy against *Pandoraea* spp., whether as a standalone approach or in combination with antibiotics.

AMPs, such as SAAP-148, LI14, CIT-8, and TM18, exert their activity primarily by permeabilizing and disrupting bacterial membranes, resulting in rapid cell death and effective biofilm eradication. They target key membrane components, including phosphatidylglycerol, cardiolipin, and LPS, leading to the dissipation of the proton motive force and leakage of vital cellular contents. Importantly, many AMPs not only inhibit biofilm formation but also eradicate mature biofilms, even in MDR strains, highlighting their therapeutic promise. Furthermore, their unique membrane-targeting mechanism makes them less likely to induce bacterial resistance, as circumventing this activity would compromise cell viability [46–50]. Collectively, these findings underscore the significance of AMPs in addressing AMR) and biofilm-associated infections, while also encouraging further research into novel peptide-based therapeutics targeting challenging pathogens such as *Pandoraea* spp.

On the other hand, efflux pumps are pivotal in drug extrusion and biofilm development, making them attractive targets for efflux pump inhibitors (EPIs), which hold promise in restoring antibiotic efficacy against bacterial infections [51]. Notably, *Staphylococcus aureus* employs the NorA efflux pump to resist multiple antibiotics and biocides. Although several EPIs have been identified, none have received clinical approval due to toxicity concerns [52]. Screening of approximately 1200 approved drugs identified nilotinib, a tyrosine kinase inhibitor, as a potent NorA EPI that synergizes with ciprofloxacin, effectively reducing both biofilm formation and mature biofilms at clinically achievable concentrations [52]. Furthermore, another study demonstrated that boeravinone B acts as a potent NorA efflux pump inhibitor, enhancing the activity of ciprofloxacin against *Staphylococcus aureus*, including methicillin-resistant strains, while significantly reducing biofilm formation and bacterial invasion into macrophages. Mechanistic assays confirmed its efflux inhibition and accumulation effects, and boeravinone B also inhibited human P-glycoprotein, underscoring its dual role in reversing bacterial resistance and modulating drug transport [53]. Interestingly, Carbonyl cyanide p-nitrophenylhydrazone (2e) exhibited synergistic antibiofilm activity with ofloxacin against MRSA, significantly lowering Minimum Biofilm Eradication Concentration values and more effectively reducing bacterial load *in vivo* than 2e alone. This synergy operates through 2e’s inhibition of the NorA efflux pump and down-regulation of quorum-sensing and virulence genes, including agrA, sarA, icaA, and hla, thus enhancing the bactericidal effect of the antibiotic [54].

In addition, metallic nanoparticles present a promising strategy to inhibit efflux pumps, potentially restoring antibiotic activity and reducing microbial biofilm formation when used in conjunction with conventional drugs [55]. Specifically, metal-based nanoparticles demonstrate significant potential to disrupt efflux activity, diminish biofilm formation, and enhance antimicrobial efficacy; however, further mechanistic studies are required to fully capitalize on this approach [56]. Based on current literature, future research should concentrate on identifying and optimizing safe and effective EPIs, including repurposed drugs such as nilotinib and natural compounds like boeravinone B, to restore antibiotic efficacy and reduce biofilm formation, particularly against challenging pathogens like *Pandoraea* spp. Additionally, investigating metal-based nanoparticles as novel efflux pump-targeting agents could offer a complementary strategy to enhance antimicrobial effectiveness, although detailed mechanistic studies remain necessary.

Most clinically significant antibiotic resistance genes in Gram-negative bacteria reside in the accessory genome, particularly on mobile genetic elements such as plasmids, integrons, and transposons, rather than the core genome. These elements facilitate horizontal gene transfer and accelerate the dissemination of resistance traits in clinical settings [57–60]. Resistance genes are frequently mobilized by insertion sequences like IS26, integrons, and transposons, enabling their translocation between plasmids and chromosomes—a major driver of multidrug resistance. In contrast, some mechanisms such as efflux pumps and reduced outer membrane permeability are encoded by core genome genes and are universally present across bacterial species. While these core determinants contribute to intrinsic resistance, they are generally less associated with high-level acquired resistance compared to genes found on plasmids and other accessory elements [57–60].

Future efforts should prioritize experimental studies, *in vivo* models, and clinical trials to explore phage therapy, AMPs, and synergistic drug regimens. Such investigations would not only address existing knowledge gaps but also pave the way for more effective management of *Pandoraea* infections. The scarcity of comprehensive literature on this topic should motivate researchers to investigate this neglected area and contribute valuable insights to the scientific community.

## Conclusion

*Pandoraea* infections represent a significant yet overlooked threat in the landscape of MDR pathogens. This review identifies a substantial gap in available clinical and experimental data, particularly regarding targeted therapeutic approaches such as phage therapy, AMPs, and antibiotic combinations. The limited real-world evidence constrains the development of reliable treatment recommendations. Consequently, future studies must focus on the isolation and characterization of *Pandoraea*-specific bacteriophages, evaluation of novel antimicrobial agents, and robust clinical investigations. Addressing these challenges is essential not only for improving patient outcomes but also for enhancing scientific understanding of this emerging pathogen.
